# Focus on Extracellular Vesicles: Exosomes and Their Role in Protein Trafficking and Biomarker Potential in Alzheimer’s and Parkinson’s Disease

**DOI:** 10.3390/ijms17020173

**Published:** 2016-02-06

**Authors:** Laura J. Vella, Andrew F. Hill, Lesley Cheng

**Affiliations:** 1 The Florey Institute of Neuroscience and Mental Health, University of Melbourne, Parkville, VIC 3010, Australia; ljvella@unimelb.edu.au; 2 Department of Biochemistry and Genetics, La Trobe Institute for Molecular Science, La Trobe University, Bundoora, VIC 3083, Australia; l.cheng@latrobe.edu.au

**Keywords:** exosomes, extracellular vesicles, neurodegeneration, protein trafficking, protein misfolding, Alzheimer’s disease, Parkinson’s disease, biomarkers

## Abstract

Growing evidence indicates that small extracellular vesicles, called exosomes, are prominent mediators of neurodegenerative diseases such as prion, Alzheimer’s and Parkinson’s disease. Exosomes contain neurodegenerative disease associated proteins such as the prion protein, β-amyloid and α-synuclein. Only demonstrated so far *in vivo* with prion disease, exosomes are hypothesised to also facilitate the spread of β-amyloid and α-synuclein from their cells of origin to the extracellular environment. In the current review, we will discuss the role of exosomes in Alzheimer’s and Parkinson’s disease including their possible contribution to disease propagation and pathology and highlight their utility as a diagnostic in neurodegenerative disease.

## 1. Introduction

Neurodegenerative diseases including Alzheimer’s, Parkinson’s and prion disease have distinct clinical manifestations and molecular pathology however they share common features such as aggregation of disease specific proteins. These include β-amyloid (Aβ) in Alzheimer’s disease (AD), α-synuclein (α-syn) in Parkinson’s disease (PD) and the prion protein (PrP) in prion disease. Further commonalities exist, namely those observed at the anatomical level revealing spread of Aβ and α-syn is in a non-random, topographically predictable manner in the brain [1,2]. Cell-to-cell contact and passive spread were initially deemed responsible; however, more recently small extracellular vesicles, called exosomes, were proposed to play a role [3,4]. A comprehensive introduction into extracellular vesicles, including exosomes, is provided in this focus edition by Kalra *et al.* [5].

Exosomes are released into the extracellular environment by most cell types and play an important role in inter-cellular communication. In the central nervous system (CNS), exosomes can mediate neuronal and glia communication, promote neuronal repair and growth and contribute to the progression of glioblastoma and neurodegenerative disease [6,7,8,9] (for a review see [10]). Once released from the donor cell, exosomes act as discrete vesicles travelling to distant and proximal recipient cells to alter cell function and phenotype. In the case of prion disease, exosomes alter recipient cell function by transmitting infectivity and initiating a cascade of events that further spreads and propagates the disease (for a review see [3]).

The idea that exosomes may be involved in the spreading of pathology in other neurodegenerative diseases such as AD and PD has recently gained considerable attention with Aβ and α-syn postulated to spread and propagate via a similar mechanism to PrP [11,12]. Here, we review the recent literature regarding the possible roles of exosomes in AD and PD and discuss their potential in biomarker discovery.

## 2. Alzheimer’s Disease

Alzheimer’s disease is characterized by the presence of insoluble plaques and tangles composed of Aβ and hyper-phosphorylated tau (p-tau), respectively. The accumulation of Aβ has been shown to interfere with synaptic plasticity which is required for neuronal signaling and implicated in pro-apoptotic signaling leading to neuronal loss. The pathological process is slow with pre-clinical AD likely to extend for more than two decades [13]. During this period, it is speculated that there is a gradual reduction of Aβ clearance in the brain and an increase in Aβ accumulation which causes the pathological hallmarks observed in late AD.

### 2.1. Association of APP and Its Metabolites with Exosomes

The Aβ isoform 1-42 (Aβ42) has been shown to play a key role in the pathology of AD due to its ability to form both intracellular and extracellular fibrils and aggregates. Aβ is produced from the processing of the amyloid precursor protein (APP) which is synthesized in the endoplasmic reticulum (ER) and then transported through the Golgi apparatus to the trans-Golgi-network (TGN). From the TGN, APP is transported to the plasma membrane driven by TGN secretory vesicles where it is then cleaved by β- and γ-secretases [14] to produce Aβ. Aβ can also be generated in the ER and Golgi within the presence of the β- and γ-secretases causing the intracellular accumulation of Aβ [15]. γ-secretase is able to cleave APP at various positions producing a number of isoforms of monomeric Aβ ranging from 39 to 43 residues in length. The liberated Aβ peptide, in particular, Aβ42 accumulates extracellularly and overtime assembles into oligomers and protofibrils leading to amyloid plaque formation (reviewed in [16]). APP at the plasma membrane can be re-internalised back through the endocytic and recycling organelles and also via the endosomal/lysosomal degradation pathway where Aβ can be generated where γ-secretase is located [17,18]. Furthermore, APP can be directly trafficked from the Golgi to lysosomes where it is cleaved and Aβ can rapidly aggregate in lysosomes due to the low pH conditions in lysosomes [19]. As Aβ42 has a high propensity to aggregate, it may be likely that this isoform is protected from degradation in the lysosome and the aggregates could eventually be released upon stress-induced cell death [19]. In AD, the lysosomal and proteasomal systems have been found to be dysfunctional in certain areas of the AD brain when compared to healthy controls [20]. It is likely that the endosomal/lysosomal system sits at a crossroad between Aβ production and degradation thus endosomal/lysosomal dysfunction may contribute to the early pathological changes seen in AD.

An endosomal organelle that fuses with lysosomes is the multi-vesicular body (MVB), also known as the late endosome compartment, which arises from the maturation of the early endosome. It is this point where contents of the MVBs can be sent for lysosomal degradation, recycled back to the trans-Golgi network or plasma membrane. Several studies have demonstrated that Aβ peptides can be packaged into intraluminal vesicles (ILVs) within the MVBs and upon fusion of the MVB with the plasma membrane the ILVs are released into extracellular environment as exosomes [21]. The role of exosomes in AD was initially explored using APP overexpressing cell lines whereby the appearance of C-terminal fragments (CTFs) of APP and Aβ were observed in exosomes [22,23]. It is plausible that exosomes containing Aβ are released to clear and regulate rising intracellular Aβ levels. A summary of the literature demonstrating Aβ and its association with exosomes can be viewed in Table 1.

**Table 1:** Studies pertaining to amyloid-β, exosomes and Alzheimer’s disease.

Exosome Source	Findings	Transfer?	Ref.
*In Vitro* Source			
SKNSH-SY5Y cells (differentiated) expressing WT APP	APP, CTFs-APP and the amyloid intracellular domain in exosomes.	×	[22]
CHO cells expressing WT APP	CTFs-APP, Aβ and several key members of the secretase family of proteases (BACE, PS1, PS2 and ADAM10) found in exosomes.	×	[23]
N2a cells expressing human AβPP Swedish mutation	Insulin-Degrading Enzyme found in exosomes is proteolytically active and assists in the degradation of endogenous Aβ.	×	[24]
N2a and primary cultured hippocampal cells of *Prnp*^+/+^ and *Prnp*^−/−^ mice	Immobilization of Aβ oligomers occurs through binding of PrP^c^ at the surface of exosomes.	×	[25]
N2a and BV-2 cells	Exosomes promote conformational changes in Aβ to form nontoxic amyloid fibrils. Exosomes are internalized into microglia to aid in Aβ degradation.	√	[26]
Mouse primary astrocytes and neurons	Astrocyte derived exosomes contain ceramide and PAR4 which were taken up by astrocytes to promote exosome-mediated astrocyte cell death.	√	[27]
Mouse primary astrocytes and neurons	Inhibition of sphingomyelinase by GW4869 reduced levels of exosomes secretion and consequently Aβ plaque formation. Exosomes containing Aβ42 injected into brains of an AD mice model (5XFAD) instigate aggregation of Aβ.	×	[28]
BE(2)-M17D cells expressing WT tau (inducible)	Exosomes contained AD associated N-terminal phosphor-tau epitopes which was also validated in CSF samples collected from AD patients.	×	[29]
Dendritic cells transfected with Lamp2b fused to the neuron-specific rabies viral glycoprotein peptide	Exosomes loaded with siRNA targeted to BACE1 were delivered into recipient cells in culture and *in vivo* resulting in the knockdown of BACE1 and a decrease in the total β-amyloid 1–42 levels.	√	[30]
Brains of transgenic mice overexpressing human APP	APP, CTFs-APP, Aβ and several key members of the secretase family of proteases (BACE and ADAM10) were found contained in exosomes.	×	[31]
CSF collected from Cynomolgus monkeys and APP transgenic mice in addition to primary neuronal and N2a cells	CSF exosomes contained Aβ. Glycosphingolipid enriched neuronal derived exosomes were able to capture Aβ as a mode of Aβ clearance.	×	[32,33]
Mouse serum of 5XFAD mice	Increase of exosomes in serum from ceramide treated mice. Ceramide treated mice were found to display increased Thioflavin S positive plaques.	×	[34]
Human serum and plasma	Detection of elevated total tau, P-T181-tau, P-S396-tau and Aβ42 by ELISA in AD patients compared to controls.	×	[35]

Recent reports suggest exosomes, which are usually isolated from extracellular fluid, can be extracted from brain tissue [28,31,36,37,38]. Perez-Gonzalez *et al.* first described the method, using it to isolate vesicles from the brains of Tg2576 mice (transgenic mice overexpressing human APP with the K670N/M671L Swedish double mutation) [31]. They observed increased levels of full length APP, CTFs–APP fragments, and Aβ in “exosomes” from brains of Tg2576 mice compared to wild type mice [31]. Using a similar isolation method, Dinkins *et al.*, reported that exosome reduction *in vivo* is associated with lower amyloid plaque load in the 5XFAD mouse model of Alzheimer’s disease [28]. Although interesting findings, the minimal experimental requirements for the definition of exosomes [39] were not met in these studies, making it difficult to draw conclusions from the data presented [28].

There is evidence to suggest that exosomes have a protective role in neurodegeneration (reviewed in [40]). Exosomes can also deliver beneficial factors such as proteolytically active plasma membrane derived insulin degrading enzyme to assist in degrading extracellular Aβ [24]. Other data has suggested that proteins found on the surface of exosomes such as the cellular prion protein (PrP^C^), a cell membrane-bound glycoprotein, can sequester Aβ induced synaptic dysfunction in the brain suggesting that exosomes have a protective role in the brain of AD patients [25]. The addition of neuron-derived exosomes with Aβ42 was found to facilitate a rapid conformational change of Aβ into nontoxic amyloid fibrils which were internalized by microglia and delivered to lysosomes, aiding Aβ degradation [26]. This supports the observation that microglia were demonstrated to internalize secreted exosomes for degradation as seen by the accumulation of labelled exosomes found within Lamp-1 positive late endosomal or lysosomal compartments in cultured microglia cells [41].

A growing number of studies have shown that dysfunction of autophagy plays a critical role in Aβ metabolism and the pathogenesis of AD [42,43]. Autophagosomes contain Aβ cleaving enzymes in addition to Aβ [44] and more recently has been shown to mediate Aβ secretion [43]. Autophagosomes containing Aβ can fuse with MVBs to form amphisomes and potentially release their ILVs in the form of exosomes upon fusion with the plasma membrane [45] leading to the extracellular release of Aβ. The induction of autophagy by rapamycin *in vivo* has been shown to lower intracellular Aβ levels leading to improved cognition [46]. Conversely, autophagy-deficient APP transgenic mice through the knockout of autophagy-related gene 7 (Atg7) displayed an increase in intracellular Aβ accumulation which lead to neurodegeneration and cognitive impairment [43]. In the same study, extracellular Aβ was found to be reduced possibly indicating that autophagy deficiency impairs secretion of Aβ to the extracellular environment.

Ultimately, as AD progresses into its advance stages the accumulation of toxic Aβ aggregates eventually overwhelms any potential mechanism of clearance. The role of exosome remains controversial [40]; however, further research will provide insight into whether exosomes are driving the accumulation of Aβ pathology or providing a method of releasing neurotoxic Aβ, likely identifying potential therapeutics to balance Aβ metabolism.

### 2.2. Association of Tau with Exosomes

In addition to amyloid plaques, aggregation of intracellular hyperphosphorylated microtubule-associated tau protein leading to neurofibrillary tangles is a common pathological feature of AD. The spread of tau occurs after Aβ plaque deposition which is firstly observed in the hippocampus and the temporal cortex. Tau pathology spreads to various areas of the brain responsible for specific sensors or motor function and consequently patients are observed to experience loss of cognitive function (reviewed in [11]). The mechanism behind the secretion and spread of tau has achieved less attention compared to Aβ. This is most likely due to the first hypothesis that tau was released following neuronal death or via an exocytic process [47]. *In vitro* studies using tau-overexpressing lines have demonstrated tau secretion can be mediated by exosomes and passive secretion [29,48,49]. More recently, using healthy untransfected neuronal cultures including neuroblastoma Neuro2a (N2a) cells and human cortical induced pluripotent stem cells (iPSCs), less than 1% of extracellular tau was found in exosomes while the majority of tau was found to be free-floating consisting of truncated c-terminal fragments of tau. Interestingly, isolating neutrally derived blood exosomes from a total exosome preparation using immunocaptured using antibodies targeting neural cell adhesion molecule (NCAM), increased levels of p-tau, P-S396-tau and P-T181-tau were detected in AD patients compared to healthy controls [35]. Further biochemical studies are required to determine whether hyperphosphorylated tau associated with AD is found in exosomes within *in vitro* and *in vivo* models.

## 3. Parkinson’s Disease

PD is a neurodegenerative disease which can be characterised by bradykinesia, resting tremor and postural instability. In PD, the signature histopathological lesions are known as Lewy bodies (LBs). Whereas the mechanisms underlying the clinical and pathological features of PD remain to be defined, the protein α-syn clearly plays a central role in the disease process being the main component of LBs.

### Association of α-Synuclein with Exosomes

Following the identification of α-syn in biological fluids including CSF [50] and blood [51], it became clear that α-syn was not exclusively an intracellular protein. Although the mechanism of α-syn release has not been fully elucidated, a role for the mitochondrial, autophagy and endosomal pathways is apparent. Both mutation and overexpression of α-syn result in aberrant processing and degradation in these pathways leading to release of both exosome associated and free α-syn into the extracellular environment [52,53,54]. The first indication that α-syn could be externalised via exosomes was provided by Emmanouilidou *et al.*, showing that exosomes derived from the conditioned media of the SH-SY5Y cell line contained α-syn [52]. This finding has since been confirmed by others in neuronal cell culture models, primary dermal fibroblasts [53,54,55,56,57,58,59] and *in vivo* [60,61]. In addition to being found in association with exosomes, α-syn has been identified in conditioned media and CSF not exosome associated [53,57,60,62]. It has been suggested that unlike the prion protein, extracellular α-syn is predominantly free with exosomes representing a small pool of the total extracellular α-syn [58,62,63,64,65]. Further studies are required to ascertain if this is indeed the case *in vivo*.

Increasing evidence suggests that exosome formation and release maybe modulated by the autophagy-lysosome pathway (ALP), whereby autophagy induction promotes exosome release, while conditions that stimulate autophagy can inhibit exosome release [45,66]. Impairments in the ALP are hallmarks of both sporadic and genetic forms of PD [67] suggesting that exosome release may be enhanced in PD. Indeed, pharmacological modulators of ALP, such as bafilomycin, inhibit of fusion of the autophagosome to the lysosome, consequently enhancing the release of α-syn in association with exosomes [53,54,59]. Bafilomycin treatment of H4 cells transfected with α-syn reduced intracellular α-syn aggregation but increased secretion of smaller oligomers by exosomes and RAB11A-associated pathways whereas high-aggregated α-syn was secreted by membrane shedding [59]. The mitochondrial complex I inhibitor, rotenone, is an environmental toxin, known to induce PD [68]. Rotenone impairs mitochondrial function but is also suggested to impair autophagic flux and lysosomal functions [69]. When treated with rotenone, enteric neurons release a greater number of exosomes containing α-syn [58]. Whilst bafilomycin and rotenone promote exosome release, the autophagy enhancer rapamycin decreased α-syn oligomer signal in exosomes, suggesting that enhanced lysosomal activity decreases exosomes release and providing further evidence of deregulated exosome release in PD.

A number of genes linked to PD have roles in autophagic and endocytic pathways including, LRRK2, VPS35 and PARK9 with evidence slowly coming to light linking these genes to exosome biogenesis and release. LRRK2 protein is implicated in protein sorting, trafficking and autophagy and is released in association with exosomes [70,71], VPS35 is involved in endosomal trafficking [72] and PARK9 protein is a P-type transport ATPase found in MVBs [55,73] and shown to regulate exosome biogenesis [55,56]. Elevated PARK9 expression increased α-syn externalization in exosomes from SH-SY5Y and H4 cells and mouse primary cortical neurons [55,56]. Interestingly, PARK9 overexpression reduced intracellular α-syn levels in SH-SY5Ys and increased α-syn externalization in exosomes [55]. By contrast, PD patient fibroblasts with loss of PARK9 function have a decreased number of intraluminal vesicles in MVBs and diminished release of exosomes into culture media [56]. Whether PARK9-mediated externalization benefits the exporting cell by decreasing intracellular levels of α-syn but consequently confers α-syn toxicity to surrounding cells is unknown [55]. Kong *et al.* proposed that the increased export of exosome-associated α-syn may explain why surviving neurons of the substantia nigra pars compacta in sporadic PD patients were observed to over-express PARK9 [55,74].

Together, these studies suggest that α-syn is released into the extracellular environment when cellular pathways fail. The importance, however, of an exosome *versus* exosome independent pathway to α-syn spread and propagation is still unclear and an area of intense study. A summary of the literature demonstrating α-syn and its association with exosomes can be viewed in Table 2.

**Table 2:** Studies pertaining to α-synuclein, exosomes and Parkinson’s disease.

Exosome Source	Findings	Transfer?	Ref.
*In Vitro* Source			
SH-SY5Y o/e WT α-syn	α-syn is constitutively secreted in exosomes and release of exosomal α-syn is calcium dependent.	×	[52]
	Lysosomal dysfunction increases release of α-syn in exosomes and α-syn transmission to recipient cells.	√	[54]
SH-SY5Y cells (differentiated) expressing WT α-syn (inducible)	Only a small portion of secreted α-syn is in exosomes. Extracellular, not exosomal α-syn, alters recipient cell function.	×	[57]
SH-SY5Y o/e WT or A53T α-syn	α-syn in neuronal cell media and CSF is abundant in the neat supernatant, not exosome.	×	[62]
Primary neurons and H4 cells o/e WT α-syn	Autophagy regulates α-syn secretion. α-syn oligomers are located inside and outside exosomes and are more prone to internalization than exosome-free α-syn oligomers and induce greater toxicity.	√	[53]
PC12 cells o/e α-syn A30P−/+ p25α	A small fraction (3% of total secreted α-syn) of α-syn monomer is associated with exosome.	×	[63]
N2a cells WT and o/e α-syn	Exosomes catalyse aggregation of exogenous α-syn.	×	[75]
SH-SY5Y or HEK293 o/e WT α-syn with or without PARK9 expression	Elevated PARK9 expression reduces intracellular α-syn levels and increases α-syn externalization in exosomes. PARK9 regulates exosome biogenesis.	×	[55]
mouse primary neurons (o/e or KD of PARK9) and patient fibroblasts	PARK9 regulates exosome biogenesis. The amount of α-syn in exosomes correlates with PARK9 expression.	×	[56]
H4 cells o/e low or high aggregating α-syn	Low-aggregated α-syn is released by exosomes and RAB11A-associated pathways whereas high-aggregated α-syn is secreted via membrane shedding.	×	[59]
Enteric primary neurons	Rotenone increases release of α-syn containing exosomes.	×	[58]
Mouse and human plasma	α-syn in plasma L1CAM-containing exosomes is higher in PD compared to healthy controls.	×	[60]
Patient urine and CSF	LRRK2 detected urine and CSF derived exosomes.	×	[70]
Patient sera	23 exosome-associated proteins differentially abundant in PD patient sera exosomes. Did not detect an enrichment of α-syn in PD patient sera derived exosomes.	×	[76]
Patient urine	LRRK2 and DJ-1 detected in urinary exosomes. Increased DJ-1 in male PD patient urinary exosomes.	×	[71]
Human CSF N2a and Oli-neu cells	α-syn is in human CSF exosomes and located inside exosomes, not outside. Sumoylation regulates exosomal release of α-syn.	×	[61]

## 4. Use of Exosomes in Biomarker Discovery for Alzheimer’s and Parkinson’s Disease

Exosomes can be isolated from extracellular fluids including cerebrospinal fluid (CSF) [77,78], blood [79] and urine [80]. As exosomes represent an enriched source of biomolecules, including proteins and nucleic acids, they are hypothesized to provide a peripheral non-invasive biomarker for neurodegenerative diseases that would be more reliable than neat CSF, blood or urine [79].

Many groups have analyzed CSF levels of Aβ42, total tau, p-tau, α-syn as biomarkers for neurodegenerative disease [35,81,82,83,84,85,86]. Variations in CSF biomarker measurements have been observed across laboratories. Studies have shown a significant decrease in CSF Aβ42 levels [84,86] or an increase in both p-tau and Aβ42 levels [35] in AD patients. Others have shown an increase in p-tau in AD patients but no significant correlation with CSF Aβ42 levels in AD patients compared to healthy patients or non-AD dementia disorders [81]. Several studies have explored the potential use of α-syn as a PD diagnostic biomarker in CSF, but the results are inconsistent with some studies showing lower α-syn in the CSF of PD patients whereas other studies show no significant change [82,83].

Exosomes extracted from CSF were enriched in proteins derived from the brain such as microglia markers (CD11b and CD45), neuron specific markers (ENO2) and vesicle associated membrane protein 2 (VAMP2) [87]. Other proteins such as prion protein (PRNP), neurogenic locus notch homolog protein 3 (NOTCH3), apolipoprotein E (APOE) associated with neurodegenerative diseases were also enriched in CSF exosomes [87]. A significant increase of total tau and p-tau was detected in exosomal CSF samples collected from postmortem AD patients compared to controls [29]. However, the increase of total tau and p-tau was not detected in the same samples when using whole CSF owing to the observation that there was an enrichment of p-tau in the exosomal samples relative to whole CSF [29]. APP has also been detected in CSF exosomes [88] and in some cases found enriched in the supernatant rather than in the exosome fraction of CSF [87]. To our knowledge, α-syn has been found in association with CSF exosomes [64]; however, the diagnostic potential is yet to be explored.

LRRK2 and DJ-1, proteins implicated in PD, have been identified in urinary and CSF exosomes. Expression of LRRK2 in urinary exosomes was variable in clinical populations, making it difficult to assess possible LRRK2 changes between PD cases and controls [70]. The authors suggest that future studies with more samples may address some aspects of power, but it seems unlikely that the LRRK2 measurement in urine alone would provide a valuable diagnostic tool [70]. DJ-1 in urinary exosomes has been proposed as a biomarker for PD in males, although a detailed study with a larger sample size is required to validate preliminary studies [71]. Proteomic analysis of PD patient sera derived exosomes performed on grouped samples of patients with genetic and sporadic forms of PD and healthy subjects identified 23 exosome-associated proteins that were differentially abundant in PD, including the regulator of exosome biogenesis syntenin 1 [76]. These protein changes were detected despite similar exosome numbers across groups suggesting that they may reflect exosome sub populations with distinct functions or selective packaging. Based on the findings of this study, it would be worth investigating the biomarker potential of the identified proteins using a larger clinical PD cohort. There are several reports of cell-free plasma biomarkers for classifying AD patients from healthy controls [89,90,91]. Although these studies show high accuracy for predicting AD, the concern for using blood based biomarkers is the relevance to brain disease. Recently, the Goetzl laboratory undertook a different approach by using immunochemical methods to harvest and enrich for brain derived exosomes in blood using NCAM and L1 cell adhesion molecule (L1CAM) [35,92,93]. In particular, they were able to specifically analyze levels of Aβ and p-tau levels from exosomes possibly derived from the brain thus removing noise generated from a general pool of exosomes found in the bloodstream. Furthermore, 12%–17% of exosomes within the total plasma exosomal population expressed NCAM and L1CAM [35]; however, it is not conclusive whether the immunocaptured exosomes are bona-fide exosomes due to the lack of characterization published in their studies. NCAM and L1CAM are not specifically unique to the brain as they are also expressed throughout the renal system [94,95]. Using a similar approach, α-syn was detected in human plasma derived exosomes (L1-CAM antibody captured), with patient (267 PD patients) plasma exosomes containing elevated levels of α-syn relative to healthy controls (215 aged matched) [60]. Total plasma α-syn levels remained unchanged [60] and no change was found in patient sera derived exosomes [76], suggesting that L1-CAM antibodies may capture a distinct, possibly CNS derived, exosome population [60]. These immunochemical capture methods provide a novel approach to improve specificity of disease biomarkers.

The nervous system is a rich source of miRNA [96], which may reflect the onset and stages of neurodegenerative disease [97]. Although miRNA can be found in CSF, they are more abundant in blood and have greater stability. Profiling of circulating miRNA associated with AD has been performed by various groups [91,98,99] and others have shown some or no correlation with CSF biomarkers [100,101]. While most of these studies utilize free circulating miRNA, exosomes provide a protective environment for the extracellular transfer of genetic material and have been shown to be enriched with miRNA species [79,102]. Using human serum collected from AD patients and healthy controls, a panel of 16 miRNAs was found to be differentially expressed in exosomes of AD patients which correlated with other methods of diagnosis such as brain-imaging, neuropsychometric testing and APOE ε4 genotyping [103]. This demonstrates the feasibility of using exosomal miRNA as a complementary tool with other biomarkers to act as a pre-screening tool for both AD and PD. The profile of exosomal miRNA in PD patient blood has not yet been determined; however, it should be explored given reports of deregulated miRNA in PD patient blood and brain tissue [104,105,106,107,108].

In future studies, it would be interesting to determine whether brain derived exosomes contain the miRNA species detected in peripheral exosomes and whether exosomal miRNA are able to disrupt gene expression to further promote neurodegeneration.

## 5. Conclusions

### 5.1. A Role for Exosomes in Protein Propagation in Alzheimer’s and Parkinson’s Disease?

A number of studies have indicated that Aβ and α-syn exhibit a prion-like self-propagation characteristic causing these toxic proteins to further aggregate and form assemblies of small oligomers to large plaques or inclusions. The deposited proteins act as a template to instigate further progression of the disease which then spread throughout the brain. The putative spread of these neurotoxic lesions has been proposed to be mediated by exosomes. An increasing number of studies has demonstrated that the propagation of the prion protein that occurs in prion diseases, such as Creutzfeldt-Jakob disease, is via an exosomal pathway [109,110]. The infectious prion protein replicates by recruiting and converting the cellular form of the prion protein (PrP^c^) to the abnormal protein isoform (PrP^sc^). The infectious pathogen can then be incorporated into exosomes as a mode of cell-to-cell contact to spread infection through tissues of the lymphoreticular system before invading the central nervous system as reviewed in [3], raising the question—does Aβ and α-syn propagation occur via a similar mechanism?

Aβ seeds have been shown to initiate Aβ accumulation and spread in the brain through studies using APP transgenic mice and performing intracerebral injections of Aβ-rich brain material from patients with AD or from aged APP transgenic mice [111]. Aβ lesions were observed to spread throughout the neocortex and hippocampal region followed by the thalamus and septal nuclei within 12 months of the intracerebral injection of Aβ rich extract [112]. Evidence of internalization has been described by studies investigating cell-to-cell transfer of Aβ [113,114]. Differentiated SH-SY5Y cells were able to transfer tagged Aβ peptides which were internalised in co-culture experiments [114]. The internalised Aβ co-localized with lysosomes; however, the exact mechanism of transfer was not investigated [114]. The phenomenon suggests that Aβ can be transferred cell-to-cell and potentially spread the pathology throughout the brain. Furthermore, astrocytes treated with amyloid peptides were found to trigger release of pro-apoptotic exosomes containing ceramide and PAR4 (prostate apoptosis response 4) [27]. These exosomes were taken up by astrocytes to further promote apoptosis, leading to exosome-mediated astrocyte cell death and potentially contributing to neurodegeneration [27].

Other studies have demonstrated that microvesicles (MVs) can play a harmful role by contributing to the accumulation of extracellular toxic Aβ in the brain. MVs are larger plasma membrane-derived vesicles of approximately 100–1000 nm which are secreted through the outward budding of the cell surface. Together with exosomes, MVs have also been shown to play an important role in cell-to-cell communication. Joshi *et al.* demonstrated that microglia-derived MVs provided an endogenous source of lipids to promote the formation of neurotoxic Aβ42 [115]. This supports the observation that lipids are able to activate fibrillisation of monomeric Aβ [116]. To further fuel the generation of neurotoxic Aβ, activated microglia found surrounding amyloid plaques were found to internalise Aβ42 leading to aggregation of Aβ42 in association with MVs as seen by confocal microscopy [115]. In the same study, exosomes secreted from microglia were also found to contain Aβ42, significantly less, however, compared to MVs.

To demonstrate the seeding hypothesis in tauopathies, tau fibrils were transduced into tau-overexpressing cells which promoted the misfolding and fibrillisation of intracellular tau resembling neurofibrillary tangles [117]. The uptake of tau seeds was found to occur spontaneously through absorptive endocytosis whereby only a small quantity of tau fibrils was required to instigate the development of intracellular filamentous tau. Furthermore, the spread of tau through interconnected regions of the brain has also been demonstrated to induce tauopathy in tau-transgenic mice [118,119].

Fetal mesencephalic grafts transplanted into the striatum of patients with PD develop α-syn positive and ubiquitin positive Lewy bodies more than a decade after transplantation, suggesting that α-syn oligomers and pathology can transfer from host-to-graft [120,121] and that α-syn has “prion-like” activity. Indeed, exosomes are capable of transferring α-syn to recipient cells *in vitro* [52,53,54] with α-syn oligomers associated with exosomes more toxic to recipient cells compared to non-exosome associated α-syn oligomers [53]. Although non-exosome associated α-syn can be taken up by cells, exosome α-syn oligomers are more prone to internalization by recipient cells [53] with transmission to recipient cells relative to the amount of α-syn released from the donor cells [54]. If disrupted by sonication prior to incubation with recipient cells, exosomal α-syn transfer is prevented, indicating that intact exosomes are required for efficient transfer and subsequent neurotoxicity [53,54].

There is increasing evidence that interactions with lipid bilayers play a role in α-syn aggregation and the pathogenesis of PD [75,122,123]. Using Thioflavin T fluorescence to monitor aggregation kinetics, Grey *et al.* recently found that exosomes catalyze the aggregation of α-syn in a similar manner to low concentrations of preformed α-syn fibrils [75]. Aggregation of exogenous α-syn was accelerated by exosomes irrespective of whether they were derived from control cells or cells over-expressing α-syn. Lipid vesicles prepared from extracted exosome lipids accelerated aggregation, suggesting that the lipids in exosomes were sufficient for the catalytic effect to arise [75].

Identification of the mechanisms of cell-to-cell transmission of pathology-associated proteins would provide a molecular pathway that could be targeted by novel therapies with the aim of disrupting or delaying spread and the subsequent progression of disease. The importance of an exosome *versus* exosome independent pathway to Aβ and α-syn spread and propagation is still unclear and remains an area of intense study.

### 5.2. The Use of Exosomes as Therapeutics

The discovery that double-stranded RNA (RNA interference; RNAi) could silence genes by degrading mRNA in a sequence-specific manner led to investigations into the use of RNAi for therapeutic purposes [124,125]. As siRNA can be rapidly degraded, there has been substantial interest in using exosomes for the delivery of siRNA *in vivo*. Cooper *et al.* developed modified exosomes that specifically target the brain by expressing a brain-targeting peptide (rabies virus glycoprotein peptide; RVG) [126] and loaded them with α-syn siRNA. To evaluate whether this approach could decrease α-syn aggregates in the brain, mice expressing the human phosphorylation-mimic S129D α-syn were injected with RVG-exosomes loaded with siRNA α-syn. This resulted in decreased α-syn mRNA and protein levels throughout the brain seven days after injection and reductions in intraneuronal protein aggregates, including in dopaminergic neurons of the substantia nigra [126]. A similar approach using RVG-exosomes was also used to deliver siRNA targeted to knockdown BACE1, a therapeutic target for AD [30]. More recently, intracerebral administration of exosomes enriched with glycosphingolipids was able to clear extracellular Aβ resulting in a decreased deposition of Aβ in the brain of APP transgenic mice [32,33]. However, recent work from Dinkins *et al.* administrated subcutaneous ceramide injections to 5XFAD mice to increase circulating exosomes and aid exosome-mediated clearance of Aβ [34]. Although amyloid levels did not significantly change, ceramide-treated 5XFAD mice displayed an increase in cortical plaque number [34]. Further work needs to be carried out to determine whether increasing or decreasing exosome secretion is beneficial in human AD.

An exosomal-based delivery system for the antioxidant, catalase, has been tested *in vitro* and *in vivo* for models of PD [127]. Catalase loaded exosomes (generated *ex vivo*) were readily taken up by neuronal cells *in vitro* and some exosomes were detected in PD mouse brains following intranasal administration. The catalase loaded exosomes provided neuroprotective effects in an *in vivo* model of PD by decreasing neuroinflammation [127]. These novel approaches pave the way to clinical trials aimed at exosome delivery of siRNA or next generation drug delivery via exosomes to delay a range of neurodegenerative diseases including Alzheimer’s and Parkinson’s disease.

In the last decade, there has been an increasing tread of research into exosomes which has expanded into neurodegenerative diseases. The body of evidence demonstrates that exosomes play an important part in communication in the brain. Although so far only confirmed in prion disease, the consensus in the field is that exosomes function as Trojan horses facilitating the accumulation and spread of neurodegenerative disease associated protein in the brain with *in vivo* data to support this hypothesis in AD and PD forthcoming. Upon understanding the physical nature of exosomes, it may be possible to manipulate their contents to deliver therapeutic factors to delay the onset of neurodegeneration.

For further information on the basic properties of extracellular vesicles, their involvement in malignant diseases, their role in cell-to-cell communication, as drug delivery vehicles, and as stem cell-derived therapeutics, the reader is referred to the various reviews of this focus edition [5,128,129,130,131].
